# Morphometric study of the diaphragmatic surface of the liver in the human fetus

**DOI:** 10.1371/journal.pone.0227872

**Published:** 2020-01-24

**Authors:** Monika Paruszewska-Achtel, Małgorzata Dombek, Mateusz Badura, Gabriela Elminowska-Wenda, Maria Dąbrowska, Magdalena Grzonkowska, Mariusz Baumgart, Anna Szpinda-Barczyńska, Michał Szpinda

**Affiliations:** 1 Department of Normal Anatomy, The Ludwik Rydygier Collegium Medicum in Bydgoszcz, The Nicolaus Copernicus University in Toruń, Bydgoszcz, Poland; 2 Department of Orthodontics, Medical University of Warsaw, Warszawa, Poland; University of Glasgow, UNITED KINGDOM

## Abstract

This study aimed to examine age-specific reference intervals and growth dynamics of the best fit for liver dimensions on the diaphragmatic surface of the fetal liver. The research material consisted of 69 human fetuses of both sexes (32♂, 37♀) aged 18–30 weeks. Using methods of anatomical dissection, digital image analysis and statistics, a total of 10 measurements and 2 calculations were performed. No statistical significant differences between sexes were found (*p*>0.05). The parameters studied displayed growth models that followed natural logarithmic functions. The mean value of the transverse–to–vertical diameter ratio of the liver throughout the analyzed period was 0.71±0.11. The isthmic ratio decreased significantly from 0.81±0.12 in the 18–19th week to 0.62±0.06 in the 26–27th week, and then increased to 0.68±0.11 in the 28–30th week of fetal life (*p*<0.01). The morphometric parameters of the diaphragmatic surface of the liver present age-specific reference data. No sex differences are found. The transverse–to–vertical diameter ratio supports a proportionate growth of the fetal liver. Quantitative anatomy of the growing liver may be of relevance in both the ultrasound monitoring of the fetal development and the early detection of liver anomalies.

## Introduction

For the last 20 years, the advancement in ultrasound imaging techniques from 2D into 3D has considerably improved the accuracy of measurements. This results in an increased precision of the monitoring of fetal development [1, 2], detectability of congenital defects [3, 4], and improved accuracy of the assessment of fetal birth weight [5–7]. However, morphometric anatomical studies are still the best determinants of the accuracy of diagnostic imaging. As emphasized by Archie et al. [8], anatomical studies are indispensable for defining whether a morphometric parameter is within the normative values. According to some authors [9–11], normative age-specific morphometric values of the growing liver in the fetus are of great importance not to the liver alone, but while assessing the whole fetal growth. Measurements of the length of the liver are particularly conducive in the detection of fetal liver pathologies, as hemangioendothelioma, hepatoblastoma and hamartoma, as well as hepatomegaly, infections, anemias, metabolic disorders and Beckwith-Wiedemann syndrome [12]. A reduction in the liver size can be caused by the intrauterine growth retardation. Contrariwise, an increase in the liver size is typical of erythroblastosis fetalis [13], falciform anemia, thalassemia or abnormalities in the transport of oxygen by hemoglobin Barts disease [9, 14, 15], maternal diabetes [3], intrauterine growth retardation, twin-to-twin transfusion syndrome (TTTS), and Down’s syndrome [16]. According to Fleischer et al. [17], the size of the liver is also a considerable clinical indicator in pregnancy complicated by Rh incompatibility. Thus, the quantitative and qualitative data is a useful reference for ultrasound liver examinations [18]. Morphometric results of individual organs obtained due to anatomical dissection with the use of innovative imaging techniques are objective in prenatal examinations and in the monitoring of pregnancy using ultrasound [19]. It is also noteworthy that the existing professional literature on the liver growth has been devoid of reports presenting values of individual parameters established for narrow age ranges, e.g. for 1–2 weeks.

Therefore, in the present study with the use of digital image analysis we aimed to quantitatively evaluate the liver diaphragmatic surface in human fetuses for consecutive gestational weeks18–30, so as to achieve its objective age-specific numerical data and growth dynamics.

The following four objectives were set:

1. to perform morphometric analysis of the diaphragmatic surface of the liver (lobe transverse and vertical diameters, isthmic diameter, right and left oblique diameters, lobe circumference, total liver circumference) in order to obtain normative values for each examined week of fetal life;
2. to establish possible differences between sexes for the analyzed parameters;
3. to compute growth dynamics for the analyzed parameters, expressed by regression curves best matched for fetal age;
4. to establish some reciprocal relationships between examined parameters.

## Material and methods

The morphometric examinations were carried out between the 1^st^ of April 2013 and 31^st^ of December 2013, at the Department of Anatomy of Ludwik Rydygier Collegium Medicum of Nicolaus Copernicus University in Toruń. The study material encompassed a group consisting of 69 human fetuses of both sexes (32♂, 37♀) aged 18 to 30 weeks of gestation of Caucasian origin. All specimens have been collected and stored in our Department of Normal Anatomy. The fetuses were obtained in years 1989–1999 from spontaneous miscarriages and preterm deliveries. On macroscopic examination, both internal and external conspicuous anatomical malformations, including those related to chromosomal disorders, were ruled out in all included specimens, thus being identified as normal. Furthermore, the fetuses studied could not suffer from growth retardation, as the correlation between the gestational age based on the crown-rump length (CRL) and that calculated by the last menstruation attained the value R = 0.98 (*p*<0.001). The study was approved by the Bioethics Committee of Nicolaus Copernicus University in Toruń (KB 161/2013). The fetal ages were determined on the crown–rump length due to the tables provided by Iffy et al. [19–29], and the known date of the beginning of the last maternal menstrual period.

The fetuses fixed in 10% neutral formalin solution were subjected to conventional anatomical dissection. The liver was visualized by median and transverse laparotomies. No macroscopic structural abnormalities in the liver structure were found. In order to precisely position and measure the liver diaphragmatic surface, each liver must have been placed on a special table for anatomical documentation in a standardized position, at which:

- the liver occupied the very center of the field of vision,
- the liver was fixed to the table with the use of pins,
- the scale was sited in parallel to the camera lens plane at the widest transverse diameter of liver,
- the measured plane of the liver was always perpendicular to the lens optical axis, maintaining a constant distance of approximately 48 cm from the latter, and finally
- the contours of the liver diaphragmatic surface must always have been acutely identified, before a picture was taken.

**Fig 1:**
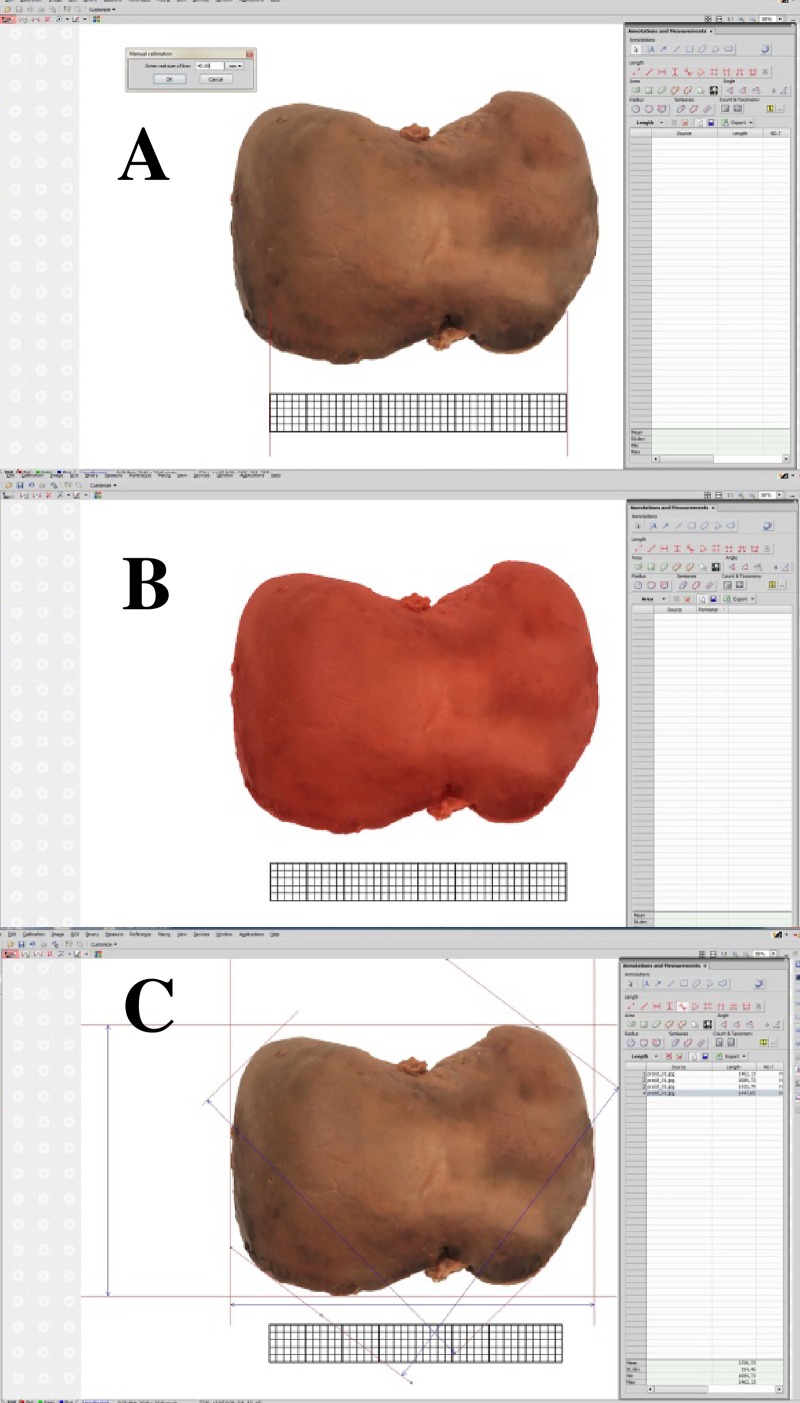
A screen of digital image analysis of NIS Elements AR 3.0 (Nikon) during: (A) calibration procedure, (B) and (C) linear measurements of the liver in the anterior projection.

The photographs were taken twice with the use of a digital SLR camera Canon 550D with a Canon 50 mm f/5.6 lens. The photographic documentation was subjected to digital image analysis using the NIS–Elements AR 3.0 software (Nikon). In order to take measurements in actual units, a calibration procedure using a millimeter scale was done (Fig 1A). The measurements were determined by selecting the extreme points of the measured distance with a cursor, and the results were added automatically to the results window (Fig 1B and 1C).

**Fig 2:**
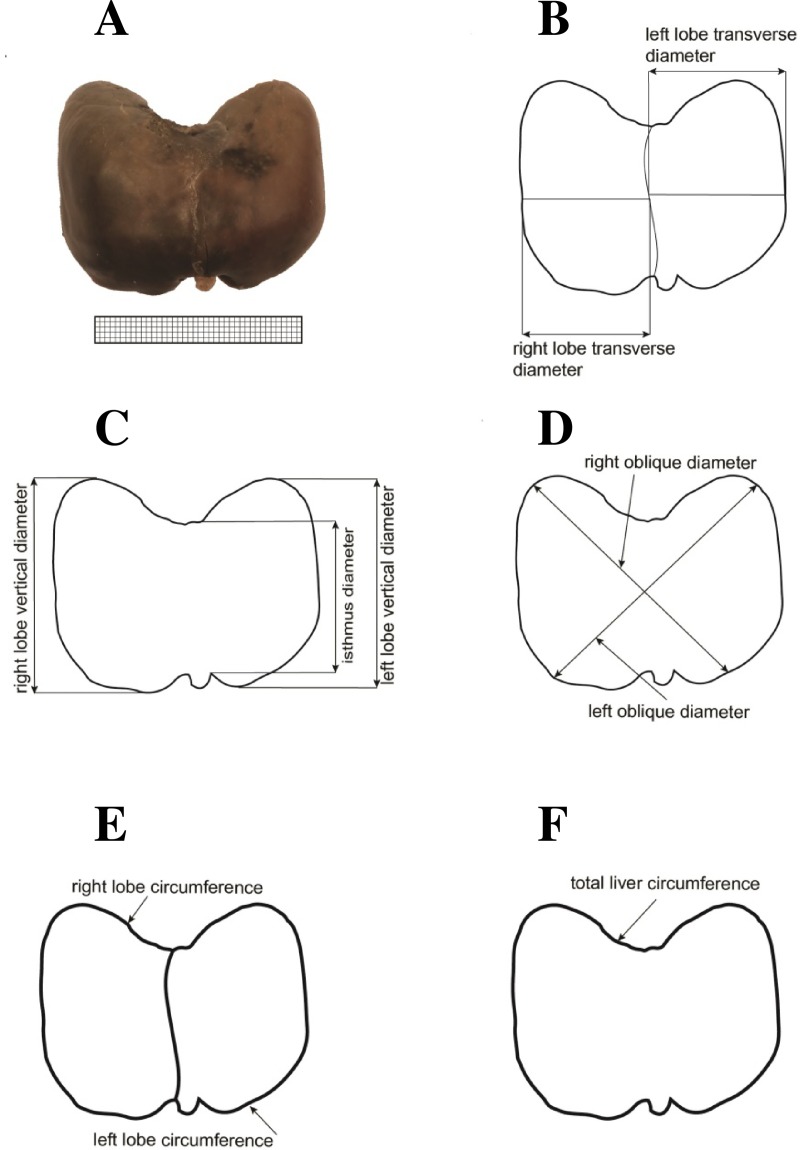
Measurements of the liver in the anterior projection: (A) diaphragmatic surface, (B) transverse diameters, (C) vertical and isthmic diameters, (D) right and left oblique diameters, (E) circumferences of the right and left lobes, (F) total liver circumference.

The obtained photographic documentation was critical for the determination of the 10 following analyzed parameters (Fig 2A–2F).

1. Transverse diameter of the right lobe, corresponding to the greatest horizontal distance between the right borderline of the liver and the falciform ligament of liver.
2. Transverse diameter of the left lobe, corresponding to the greatest horizontal distance between the falciform ligament of liver and its left borderline.
3. Vertical diameter of the right lobe, corresponding to the greatest vertical distance, between the superior and inferior borderlines of the right lobe.
4. Vertical diameter of the left lobe, corresponding to the greatest vertical distance between the superior and inferior borderlines of the left lobe.
5. Right oblique diameter of the diaphragmatic surface, corresponding to the longest oblique distance of the liver between its uppermost point of the right borderline to its lowermost point of the left borderline.
6. Left oblique diameter of the diaphragmatic surface, corresponding to the longest oblique distance of the liver between its uppermost point of the left borderline to its lowermost point of the right borderline.
7. Isthmic diameter of the diaphragmatic surface of the liver, corresponding to the shortest vertical distance between its superior and inferior borders.
8. Circumference of the right lobe.
9. Circumference of the left lobe.
10. Total circumference of the diaphragmatic surface of the liver.

Based on the obtained measurements, the following two indexes were calculated:

1. transverse–to–vertical diameter ratio, corresponding to a quotient of the transverse diameter to the vertical diameter of the right lobe,
2. isthmic ratio, corresponding to a quotient of the isthmic diameter to the vertical diameter of the right lobe.

In fact, all livers were recorded and measured twice by two independent researchers (M.P.A., M.B.) after a-5-day interval. Each measurement was performed three times under the same conditions but at different times, and then averaged. For clarity, the differences between repeated measurements, as the intra-observer variation were assessed by the one–way ANOVA with post-hoc RIR Tukey test. Thus, in order to examine the inter-observer reproducibility, the intra-class correlation coefficients (ICC) were calculated. The results were statistically analyzed. Distribution of variables was checked using the Shapiro-Wilk (W) test. The homogeneity of variance was checked using Fisher's test. As the distribution of the analyzed variables proved to be normal, the results were expressed as arithmetic means with standard deviations (SD). The characterization of growth dynamics of the analyzed parameters was based on linear and non–linear regression analysis. The match between the estimated curves and measurement results was evaluated based on the coefficient of determination (R^2^). The calculations were done using the STATISTICA 12.5 and PQStat 1.6.2. software.

## Results

Table 1 lists the characteristics of the study group, including their number, age and sex.

**Table 1:** Age, number and sex of the fetuses examined.

Gestational age	Crown-rump length (mm)				Number of fetuses	Sex	
Weeks	Mean	SD	Min.	Max.		♂	♀
18	138.3	5.5	131.0	143.0	4	3	1
19	151.5	3.6	145.0	155.0	6	4	2
20	162.4	3.2	159.0	167.0	7	3	4
21	173.7	3.6	170.0	180.0	7	5	2
22	185.2	3.1	181.0	190.0	6	1	5
23	199.5	3.9	195.0	204.0	6	4	2
24	210.0	3.8	205.0	214.0	10	2	8
25	216.0	2.2	215.0	220.0	5	2	3
26	230.0	4.6	225.0	233.0	3	1	2
27	239.5	3.1	235.0	242.0	4	2	2
28	251.0	2.6	247.0	253.0	7	1	6
30	264.0	1.2	263.0	265.0	4	4	0
Total					69	32	37

No statistically significant differences were found in assessing the intra-observer reproducibility of liver measurements (*p*>0.05). As displayed in Table 2, the intra-class correlation coefficients (ICC) calculated on the base of two independent observers were statistically significant (*p*<0.001) and of excellent reproducibility. As presented in Table 3, the statistical analysis of the 10 analyzed parameters did not reveal sex dimorphism (*p*>0.05). Therefore, each parameter was aggregately examined for the whole group without taking the sex into account (Tables 4 and 5).

**Table 2:** Intra–class correlation coefficient (ICC) values for inter–observer reproducibility.

Parameter	ICC (2,1)
right lobe transverse diameter	0.991*
left lobe transverse diameter	0.988*
right lobe vertical diameter	0.971*
left lobe vertical diameter	0.945*
isthmus diameter	0.967*
right oblique diameter	0.974*
left oblique diameter	0.989*
right lobe circumference	0.979*
left lobe circumference	0.986*
total liver circumference	0.979*

Intra-class correlation coefficients marked with * are statistically significant at p<0.001.

**Table 3:** Statistical analysis of liver morphometric parameters (in mm) in both sexes of three age ranges.

Liver parameters	Fetal age of 18–21 weeks					Fetal age of 22–25 weeks					Fetal age of 26–30 weeks				
	male (n = 15)		female (n = 9)		p	male (n = 9)		female (n = 18)		p	male (n = 8)		female (n = 10)		p
	mean	SD	mean	SD		mean	SD	mean	SD		mean	SD	mean	SD	
right lobe transverse diameter	17.21	3.33	19.47	3.43	0.125	22.66	4.70	24.79	4.62	0.274	27.85	5.62	27.29	3.86	0.808
left lobe transverse diameter	15.51	4.48	15.08	3.62	0.812	21.94	2.67	19.33	4.22	0.104	22.73	4.30	23.66	5.10	0.687
right lobe vertical diameter	22.70	3.81	24.83	5.19	0.260	31.94	6.08	30.84	5.60	0.643	36.26	6.31	33.87	5.16	0.389
left lobe vertical diameter	19.21	4.57	21.13	4.84	0.342	25.04	3.93	25.02	5.59	0.992	30.18	8.12	23.12	4.00	0.528
isthmic diameter	17.90	3.47	18.87	4.72	0.568	22.64	3.79	21.21	4.20	0.397	25.65	7.33	21.34	2.91	0.107
right oblique diameter	31.02	4.31	33.89	4.91	0.148	42.68	4.59	42.07	5.22	0.771	47.85	5.19	44.39	4.53	0.151
left oblique diameter	32.05	5.56	35.73	5.61	0.132	45.09	5.87	44.75	5.47	0.884	51.34	6.71	51.23	7.02	0.973
right lobe circumference	77.26	14.28	80.94	15.12	0.555	101.47	14.82	104.56	14.84	0.615	119.24	21.57	113.40	13.31	0.490
left lobe circumference	67.34	12.06	68.49	12.90	0.827	84.63	11.34	84.09	14.85	0.372	544.40	124.58	432.81	111.80	0.063
total liver circumference	104.94	22.55	109.01	18.58	0.653	135.95	13.75	141.13	19.82	0.490	162.52	22.66	155.99	20.49	0.530

Note: No statistically significant differences between sexes.

**Table 4:** Numerical data of the growing liver.

Diaphragmatic surface (mm)											
Age [weeks]	n	right lobe transverse diameter		left lobe transverse diameter		right lobe vertical diameter		left lobe vertical diameter		isthmic diameter	
		Mean	SD	Mean	SD	Mean	SD	Mean	SD	Mean	SD
18	4	15.38	2.00	14.07	3.96	18.91	0.74	16.18	3.38	14.66	0.20
19	6	18.16	3.08	12.26	3.39	20.44	3.37	18.04	3.90	17.52	4.56
20	7	18.60	3.74	15.52	2.93	26.71	3.96	21.75	5.22	20.45	4.50
21	7	18.95	4.05	18.55	3.98	25.54	2.89	21.88	4.07	18.78	2.42
18–21	24	18.06 ^(a)^	3.48	15.35 ^(a)^	4.10	23.50 ^(a)^	4.40	19.93 ^(a)^	4.67	18.27 ^(a)^	3.91
22	6	21.16	3.41	18.37	2.32	27.51	6.40	23.86	7.68	21.13	6.34
23	6	21.59	3.33	21.73	4.60	32.73	5.39	25.92	5.13	22.00	3.51
24	10	25.80	5.39	18.61	3.86	30.75	3.93	24.86	3.95	21.97	2.15
25	5	27.13	2.64	23.73	1.78	34.74	6.84	25.67	4.27	21.40	5.46
22–25	27	24.08 ^(b)^	4.67	20.20 ^(b)^	3.93	31.21 ^(b)^	5.67	25.02 ^(b)^	5.02	21.68^(b)^	4.05
26	3	27.56	1.02	20.23	1.81	30.12	2.16	25.63	4.29	20.01	1.64
27	4	26.04	5.39	24.54	1.47	36.68	6.60	26.80	6.83	22.44	3.43
28	7	26.60	5.20	24.21	6.04	33.53	3.81	23.20	3.20	22.30	2.40
30	4	30.65	4.17	22.51	5.55	39.24	7.00	31.55	11.68	28.19	10.35
26–30	18	27.54 ^(c)^	4.58	23.24 ^(c)^	4.65	34.93 ^(c)^	5.65	26.26 ^(c)^	6.97	23.26 ^(c)^	5.61
(a) vs (b)		p < 0.001		p < 0.001		p < 0.001		p < 0.01		p < 0.05	
(a) vs (c)		p < 0.001		p < 0.001		p < 0.001		p < 0.01		p < 0.01	
(b) vs (c)		p < 0.05		p = 0.081		p = 0.093		p = 0.778		p = 0.543	

Note: Statistically significant differences in columns are marked by different letters ^a^,^b^ and ^c^.

**Table 5:** Numerical data of the growing liver.

Diaphragmatic surface (mm)											
Age [weeks]	n	right oblique diameter		left oblique diameter		right lobe circumference		left lobe circumference		total liver circumference	
		Mean	SD	Mean	SD	Mean	SD	Mean	SD	Mean	SD
18	4	27.21	3.60	28.39	4.89	69.47	14.89	64.41	16.37	101.92	31.58
19	6	29.69	1.93	29.03	2.95	71.29	7.86	59.73	7.56	91.79	4.85
20	7	33.92	5.12	36.84	4.78	82.86	14.42	71.99	12.12	110.96	18.30
21	7	35.12	3.34	36.66	4.76	85.96	15.15	72.38	10.77	117.15	20.59
18–21	24	32.10 ^(a)^	4.66	33.43 ^(a)^	5.75	78.64 ^(a)^	14.39	67.78 ^(a)^	12.11	106.46 ^(a)^	20.83
22	6	38.27	3.28	39.80	3.19	90.78	7.09	79.83	16.25	124.38	10.05
23	6	42.66	5.37	44.65	4.04	99.69	12.07	85.42	16.94	136.20	17.36
24	10	41.58	4.11	44.48	4.75	105.31	10.10	81.89	10.71	139.10	13.58
25	5	48.02	2.08	51.95	3.28	119.86	17.54	92.98	10.33	161.85	13.64
22–25	27	42.28 ^(b)^	4.94	44.86 ^(b)^	5.49	103.53 ^(b)^	14.62	84.27 ^(b)^	13.56	139.40 ^(b)^	17.92
26	3	45.31	0.65	46.38	1.64	104.39	6.15	82.75	11.97	142.46	3.02
27	4	44.26	5.02	52.82	7.03	115.83	18.88	94.59	13.01	160.70	25.70
28	7	44.74	5.19	50.58	7.82	112.43	10.45	93.80	18.43	156.70	19.32
30	4	50.13	5.66	54.64	6.00	131.10	24.46	104.72	17.78	173.26	23.38
26–30	18	45.92 ^(c)^	5.01	51.28 ^(c)^	6.68	116.00 ^(c)^	17.15	94.56 ^(c)^	16.48	158.90 ^(c)^	21.09
(a) vs (b)		p < 0.001		p < 0.001		p < 0.001		p < 0.001		p < 0.001	
(a) vs (c)		p < 0.001		p < 0.001		p < 0.001		p < 0.001		p < 0.001	
(b) vs (c)		p = 0.070		p < 0.01		p < 0.05		p = 0.075		p < 0.05	

Note: Statistically significant differences in columns are marked by different letters ^a^,^b^ and ^c^.

During the prenatal development, the mean transverse diameter of the right lobe of the liver ranged from 15.38±2.00*mm* in the 18th week of fetal life to 30.65±4.17 *mm* in the 30th week of fetal life. This dimension increased following the natural logarithmic function: *y* = −67.531+28.786×*ln*(*age*)±3.652. The vertical diameter of the right lobe diaphragmatic surface ranged from 18.91±0.74 *mm* in the 18th week to 39.24±7.00 *mm* in the 30th week of fetal life, following the natural logarithmic function *y* = −82.962+35.695×*ln*(*age*)±4.359. In the analyzed period, the mean transverse diameter of the left lobe of the liver diaphragmatic surface was found to increase from 14.07±3.96 *mm* in the 18th week to 22.51±5.55 *mm* in the 30th week of fetal life. This diameter revealed an increase in accordance with the natural logarithmic function: *y* = −57.945+24.602×*ln*(*age*)±3.206. The vertical diameter of the left lobe was on average 16.18±3.38 *mm* at week 18 and 31.55±11.68 *mm* at week 30, increasing in accordance with the natural logarithmic function *y* = −43.699+21.304×*ln*(*age*)±4.538.

The mean of the right oblique diameter of the liver diaphragmatic surface ranged from 27.21±3.60 *mm* in the 18th week to 50.13±5.66 *mm* in the 30th week of fetal life. This dimension increased with age following the natural logarithmic function: *y* = −95.919+43.146×*ln*(*age*)±3.993. The mean left oblique diameter of the projection of the liver diaphragmatic surface ranged from 28.39±4.89 *mm* in the 18th week to 54.64±6.00 *mm* in the 30th week of fetal life, increasing with age in accordance with the natural logarithmic function: *y* = −125.551+53.533×*ln*(*age*)±4.769.

During the prenatal development, the isthmic diameter of the liver diaphragmatic surface grew from 14.66±0.20 *mm* at week 18 to 28.19±10.35 *mm* at week 30. Due to a considerable spread of the obtained values, it was impossible to match its growth model.

**Fig 3:**
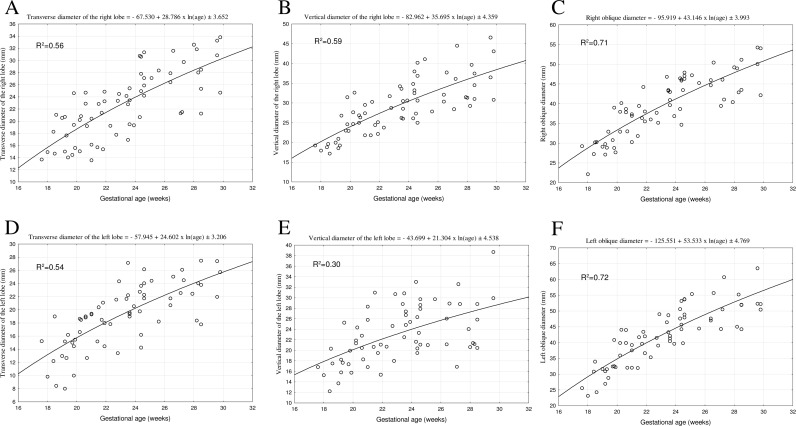
The best-fit regression formulae for the diaphragmatic surface of the fetal liver: transverse diameter, vertical diameter, right and left oblique diameter, vertical diameter of the right and left lobe.

**Fig 4:**
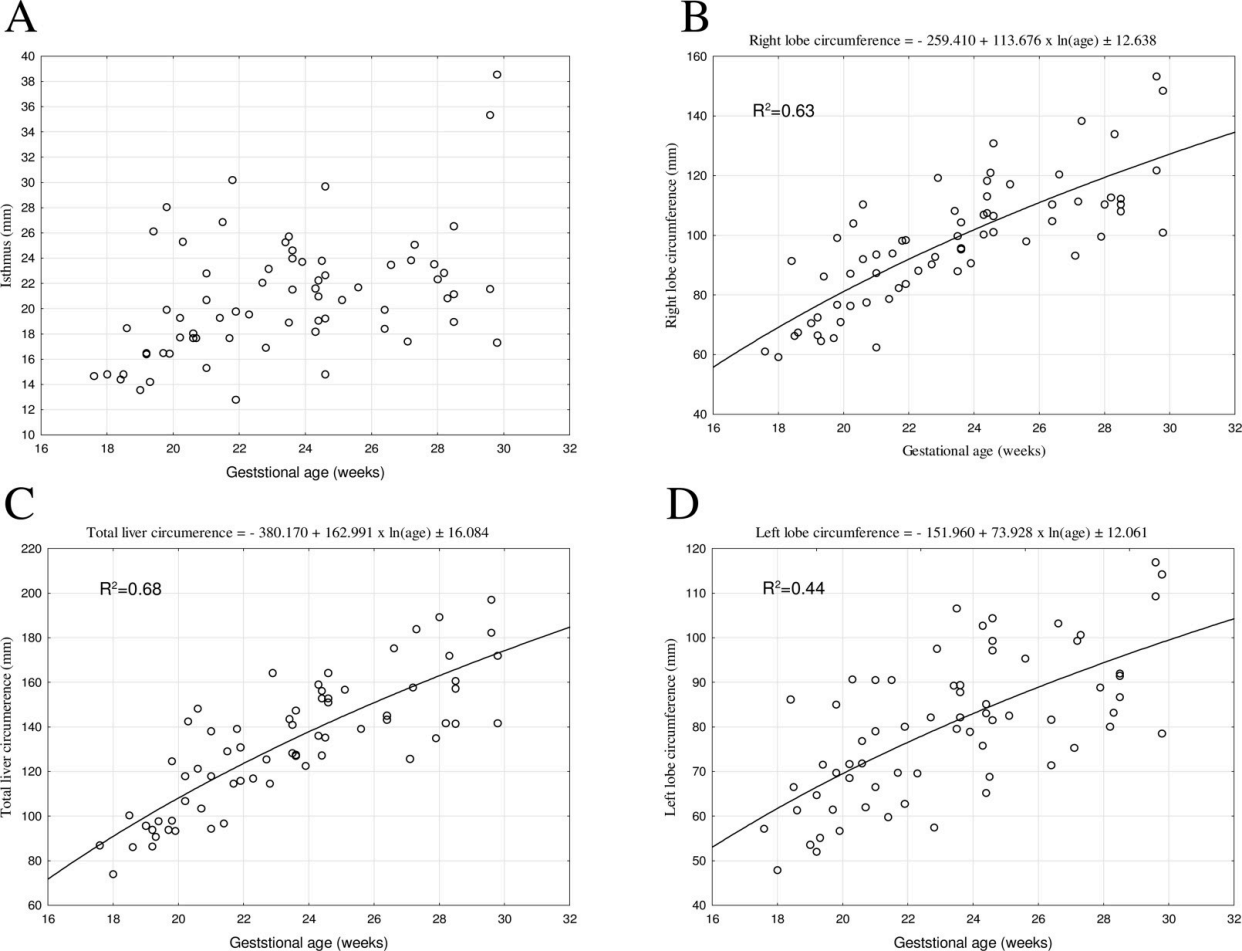
The best-fit regression formulae for the diaphragmatic surface of the fetal liver: isthmus, right and left lobe circumference, total circumference.

In the analyzed period, the mean circumference of the right lobe varied from 69.47±14.89 *mm* in the 18th week to 131.10±24.46 *mm* in the 30th week of fetal life. An increase in circumference at that period followed the natural logarithmic function: *y* = −259.410+113.676×*ln*(*age*)±12.638. The mean circumference of the left lobe of the liver diaphragmatic surface was found to grow from 64.41±16.37 *mm* in the 18th week to 104.72±17.78 *mm* in the 30th week of fetal life, and modelled the natural logarithmic function: *y* = −151.960+73.928×*ln*(*age*)±12.061. The mean total circumference of the projection of the liver diaphragmatic surface ranged from 101.92±31.58 *mm* in the 18th week to 173.26±23.38 *mm* in the 30th week of fetal life. An increase in circumference at that period followed the natural logarithmic function: *y* = 380.170+162.991×*ln*(*age*)±16.084. All growth models expressed by the estimated curves best-matched to the measurement results with their coefficients of determination (R^2^) have been shown in Figs 3 and 4.

In the analyzed age ranges, the mean values of the transverse–to–vertical diameter ratio on the diaphragmatic surface of the liver did not differ in a statistically significant manner (*p* = 0.732) and reached the mean value of 0.71±0.11*mm*.

The isthmic ratio decreased significantly from 0.81±0.12 *mm* in the 18–19th week to 0.62±0.06 *mm* in the 26–27th week, and then increased to 0.68±0.11 *mm* in the 28–30th week of gestation (*p*<0.01).

## Discussion

After reviewing the professional literature, it turned out that prior to the present study, only Albay et al. [9] used the anatomical method to study the height (or length, corresponding to liver vertical diameter) and width (corresponding to liver transverse diameter) of the diaphragmatic surface of the fetal liver. Their study included four age groups, i.e. three trimesters of gestation and a group of full–term fetuses (born in the 38–40th week of gestation). In those four age subgroups, the liver heights were 13±2 *mm*, 25±8 *mm*, 41±7 *mm* and 48±8 *mm*, respectively. Of note, in each age range, the liver width was significantly greater than the liver height and reached the values 19±3 *mm*, 39±12 *mm*, 67±11 *mm* and 82±9 *mm*, respectively. These authors also demonstrated that the rate of liver growth in width and height was greater than its growth in thickness, which was closely correlated with the abdomen growth rate in the transverse, vertical and sagittal dimensions, correspondingly. In turn, Vintzileos et al. [30] examined 100 pregnant women between weeks 20 and 41 of gestation, and found the liver length until week 30 to increase less dynamically than after week 30. The mean liver lengths given by Vintzileos et al. [30] were 27.30±6.4 *mm* at week 20 and 38.70±5.00 *mm* at week 30 of gestation. Between weeks 20 and 30, the liver length grew by 1.2 mm a week, while between weeks 31 and 41, the liver length grew by 1.7 mm a week. Of note, those results correlate with ours since the vertical diameter of the right lobe of the liver on its diaphragmatic surface was 18.91±0.74 *mm* at week 18, 26.71±3.96 *mm* at week 20, and 39.24±7.00 *mm* at week 30 of gestation. However, Roberts et al. [31], based on an ultrasound examination of 350 mothers with normal pregnancy, presented a graphical correlation between the length of the fetal liver and the gestational age between weeks 15 and 40, which followed a quadratic function (*R*^2^ = 0.88), reaching its extreme at week 40. Although a detailed growth model was not presented in the aforementioned study, the analysis of included Fig 2 just indicated that it must have been a quadratic function with a negative coefficient referring to *x*^2^. In fact, this model would have exhibited a negative slope beyond week 40. To our opinion, it would be a more complete solution, suitable for a wider age range, to match a natural logarithmic function that is an increasing function by its nature, but with a gradual inhibition of growth. We performed the morphometric analysis of the diaphragmatic surface of the liver because of two reasons that may be useful and relevant in clinical practice. Firstly, the diaphragmatic surface of the liver directly contacts the undersurface of the diaphragm. This means that any potential places of low resistance in the diaphragm appear to be sealed by the diaphragmatic surface of the liver. It is noteworthy that in the fetus, a diaphragmatic hernia results in life-threatening pulmonary hypoplasia that requires *in utero* surgery. Secondly, since the fetal liver is indispensable for hepatic hematopoiesis, its right and left lobes facing the diaphragm are virtually equal in size. Possibly, the precise evolution of the liver diaphragmatic surface expressed by our age-specific reference numerical data may reflect the intensity of the hematopoiesis process. Of note, in the present study the best-fit models have indeed been natural logarithmic functions. Such a function displays a typical decrease in the liver growth rate with fetal age that is conspicuous between groups 2 and 3 for most diameters. As reported by Chang et al. [32] in fetuses aged 20–30 weeks, both the height and transverse diameter of the liver increased linearly, according to the following functions: *y* = 0.66+1.19×(*age*); (*r* = 0.58, *p*<0.001) and *y* = −15.89+2.37×(*age*); (*r* = 0.78, *p*<0.001), respectively. In a study by Phatihattakorn et al. [33] in 750 fetuses aged 13–40 weeks, the liver length increased from 2.59±2.08 to 60.06±3.49 *mm*, according to the linear regression: *y* = 1.528×(*age*)−5.676. Tongprasert et al. [34] examined 640 fetuses between 14 and 40 weeks of gestation and revealed the liver length to increase with fetal age in accordance with the linear function: *y* = 1.61×(*age*)−6.75 (*R*^2^ = 0.94, *p*<0.001), from 22.3 *mm* at week 18 to 41.6 *mm* at week 30.

In the material under examination, the circumferences of the right and left lobes on the diaphragmatic surface to increase logarithmically as follows: *y* = −259.410+113.676×*ln*(*age*)±12.638 and *y* = −151.960+73.928×*ln*(*age*)±12.061, respectively. Furthermore, the total liver circumference computed the logarithmic function: *y* = 380.170+162.991×*ln*(*age*)±16.084. It should be noticed that our earlier study on the visceral surface of the fetal liver [35] showed the circumferences of right and left lobes to grow logarithmically as well, in accordance with the following regressions, as: *y* = −287.401+120.550×*ln* (*Age*)±10.00 and *y* = −295.715+122.097×*ln* (*Age*)±10.45, respectively. The total liver circumference modelled the natural logarithmic function: *y* = −517.502+210.340×*ln* (*Age*)±13.714.

A study by Dimaano and Rivera [36] referring to the adequate age group of diabetic Filipino mothers showed liver lengths for diabetic subjects to be larger in comparison to non-diabetic mothers. Anderson et al. [37] examined 44 women with gestational diabetes and found the liver length in the fetuses to increase approximately by 1.0−1.2 *mm* per week up to week 28, and much more quickly, i.e. 1.70−1.76 *mm* per week, beyond week 28. Mirghani et al. [38] carried out a study in 123 pregnant women at 21–24 weeks of pregnancy, 19 of whom suffered from gestational diabetes, and found the height of the right lobe to be significantly greater in the diabetic mothers. Similar results regarding an increase in the height of the liver right lobe were found by Roberts et al. [39] in fetuses at risk of erythrocyte alloimmunization due to Rh incompatibility. This was because the liver was the locale of extramedullary hematopoiesis. In 93% of the fetuses with diagnosed anemia, liver length was above the 95th percentile, while in the remaining 7% of fetuses, liver length was within the upper limit of normality, above the 75th percentile. Roberts et al. [31] demonstrated that hepatomegaly is a useful indicator of increased hepatic hematopoiesis in Rh–immunized pregnancy. This fact was confirmed by statistically significant (p < 0.001) high correlation coefficients both between liver length and fetal hemoglobin concentration (*r* = −0.794; the authors erroneously described the correlation as positive, *r* = 0.794) and between liver length and the number of reticulocytes (*r* = 0.721). In all fetuses with hemoglobin levels below 100*mg*/*L*, liver length exceeded the 90th percentile. The aforementioned Vintzileos et al. [30], Anderson et al. [34] and Roberts et al. [31] emphasized the need for further studies of liver development, especially in mothers with gestational diabetes or anemia.

Not previously covered by the professional literature liver morphometric parameters with respect to its diaphragmatic surface have been determined in the present study, namely: transverse, oblique and isthmic diameters and circumferences of the lobes. Our study demonstrated that the developmental dynamics of these parameters increased in accordance with natural logarithmic functions.

The main limitation of the present study is a small number of examined fetuses in particular gestational weeks. Another weakness is a lack of fetuses younger than 18 weeks and older than 30 weeks of gestation.

## Conclusions

1. The morphometric parameters of the diaphragmatic surface of the liver present age-specific reference data.
2. No sex differences are found in numerical data of morphometric parameters on the diaphragmatic surface of the fetal liver.
3. All the parameters studied follow natural logarithmic functions.
4. The transverse–to–vertical diameter ratio supports a proportionate growth of the fetal liver.
5. Quantitative anatomy of the growing liver may be of relevance in both the ultrasound monitoring of the fetal development and the early detection of liver anomalies.
